# Believability of messages about preventing breast cancer and heart disease through physical activity

**DOI:** 10.1186/s40359-018-0213-8

**Published:** 2018-01-18

**Authors:** Tanya R. Berry, Kelvin E. Jones, Kerry S. Courneya, Kerry R. McGannon, Colleen M. Norris, Wendy M. Rodgers, John C. Spence

**Affiliations:** 1 Faculty of Kinesiology, Sport, and Recreation, University of Alberta, Edmonton, AB T6G 2H9 Canada; 2 School of Human Kinetics, Laurentian University, Sudbury, Canada; 3 Faculty of Nursing, University of Alberta, Edmonton, Canada

**Keywords:** heart disease, breast cancer, prevention messages, believability, dual processing

## Abstract

**Background:**

The purpose of this research was to examine the relationships of self-reported physical activity to involvement with messages that discuss the prevention of heart disease and breast cancer through physical activity, the explicit believability of the messages, and agreement (or disagreement) with specific statements about the messages or disease beliefs in general.

**Methods:**

A within subjects’ design was used. Participants (*N* = 96) read either a breast cancer or heart disease message first, then completed a corresponding task that measured agreement or disagreement and confidence in the agreement or disagreement that 1) physical activity ‘reduces risk/does not reduce risk’ of breast cancer or heart disease, 2) that breast cancer or heart disease is a ‘real/not real risk for me’, 3) that women who get breast cancer or heart disease are ‘like/not like me’, and 4) that women who get breast cancer or heart disease are ‘to blame/not to blame’. This task was followed by a questionnaire measuring message involvement and explicit believability. They then read the other disease messages and completed the corresponding agreement and confidence task and questionnaire measures. Lastly, participants completed a questionnaire measuring physical activity related attitudes and intentions, and demographics.

**Results:**

There was no difference in message involvement or explicit believability of breast cancer compared to heart disease messages. Active participants had a higher confidence in their agreement that physical activity is preventive of heart disease compared to breast cancer. Multinomial regression models showed that, in addition to physical activity related attitudes and intentions, agreement that physical activity was preventive of heart disease and that women with heart disease are ‘like me’ were predictors of being more active compared to inactive. In the breast cancer model only attitudes and intentions predicted physical activity group.

**Conclusions:**

Active women likely internalized messages about heart disease prevention through physical activity, making the prevention messages more readily available within memory, and active women may therefore process such information differently. The study of how health-related beliefs are created and are related to perceptions of prevention messages is a rich area of study that may contribute to more effective health promotion.

## Background

Physical activity is preventive of both breast cancer [1] and heart disease [2], and is therefore promoted by breast cancer and heart disease foundations. The work presented here investigated the believability of messages that promote physical activity as preventive of heart disease and breast cancer and how believability may be related to physical activity related attitudes, intentions, and behavior. Believability refers to perceptions of how likely, convincing, or trustworthy messages are; that is, whether the message is believed and accepted, or read with skepticism [3]. Researchers have examined the believability of health promotion messages as a precursor to attitudes and related constructs. For example, O’Cass and Griffin [4] found that the believability of anti-smoking advertisements was predictive of attitudes. Others have shown that believability of graphic images on cigarette packages is related to increased risk perception and reduced desire to smoke [5], that the majority of students disbelieved messages to reduce binge drinking and disbelief was related to drinking more alcohol and reporting more hangovers than those who believed the message [6], and that believability of hand washing statistics is related to attitudes toward the behavior [7]. Thus, there is evidence that general believability of health messages is related to attitudes and should be considered when examining health promotion or prevention messages.

Believability can also be related to specific aspects of a message, and may be automatically activated. In such cases, the ‘belief’ is not easily controlled and the measurement of the construct is considered implicit [8]. Huang and Hutchinson [8] developed a task that measures beliefs that automatically arise from existing memory associations after exposure to related information. They found their measure predicted attitudes toward commercial products and toward a health topic (hepatitis C prevention). Others used this task and found that automatically activated beliefs and explicit believability of exercise-for-health messages were correlated but automatically activated and explicit believability of exercise-for-appearance messages were not. The automatically activated belief of appearance messages was negatively related to intentions to exercise [9]. The appeal of the task created by Huang and Hutchinson is that it has the potential to provide additional information about cognitive responses to specific elements within persuasive messages [8]. However, it may also be that associations exist due to messages repeatedly heard from other sources, such as the media. Given this possibility, this research examined responses to statements specific to the readings used in the research, and responses to statements that reflect how breast cancer or heart disease are commonly perceived or represented.

It has been shown that women feel heart disease is more preventable and controllable than breast cancer and that breast cancer poses a greater risk than heart disease [10]. Thus, heart disease prevention messages should be more believable because of stronger associations between prevention behaviors and heart disease that have been created through learning [8]. There should also be stronger agreement with statements of risk for breast cancer compared to heart disease [10]. Further, although breast cancer gets a disproportionate amount of media coverage compared to heart disease, heart disease media messages emphasize prevention and lifestyle behaviors such as physical activity, while breast cancer media discusses ‘survivorship’ and screening behaviors such as mammograms [11]. There may, therefore, be stronger agreement with a message that physical activity is preventive of heart disease compared to breast cancer. Given that heart disease is considered controllable through lifestyle behaviors and that media emphasizes prevention, women who do not adhere to prevention behaviors such as physical activity may be blamed for getting heart disease. Because physical activity messages draw the attention of people who are already active and already have a positive attitude toward physical activity [12], physical activity status may moderate the effects of prevention messages.

O’Cass and Griffin [4] reported that the explicit believability of anti-smoking and anti-binge drinking messages was a significant predictor of related attitudes. They also found that involvement with a topic was predictive of believability and attitudes, and that attitudes predicted intentions. They used Zaichowsky’s [13] definition and measure that operationalizes involvement as personal relevance based on values and interests. Thus, the current research also examined if involvement with heart disease and breast cancer prevention messages was related to believability, attitudes, intentions, and behavior. Further, affective attitudes were assessed as a predictor of physical activity, rather than instrumental attitudes, because affective attitudes are more strongly related to physical activity behavior [14].

The purpose of this research was to examine the relationships of message involvement, the general believability of messages that discuss physical activity as preventive of heart disease and breast cancer, automatically activated agreement (or disagreement) with specific statements related to the messages or diseases (i.e., automatically activated believability), and physical activity-related attitudes and intentions to self-reported physical activity. The specific statements represented the message read during this research (i.e., that physical activity reduces risk of breast cancer or heart disease), reflected more general perceptions of risk for breast cancer and heart disease [10], reflected media messages that discuss ‘typical’ women who have breast cancer or heart disease, or that women who do not engage in preventive behaviors are to blame for their disease [11]. It is hypothesized that 1) message involvement will be positively related to automatically activated and explicit believability; 2) automatically activated and explicit believability will be positively related to physical activity-related behavior; 3) more active women will show stronger message involvement, explicit believability, and automatically activated beliefs, and 4) the relationships outlined in the first three hypotheses will be stronger for heart disease than breast cancer.

## Methods

### Participants

One hundred women aged 18 to 80 years (M = 33.05, [SD = 16.25]) were recruited using posters and e-mail list-serves from a university campus community, a senior’s club, and a religious institution. Given that participants contacted the researchers if they wanted to participate after seeing recruitment information, it is not possible to determine response rate.

### Materials

#### Messages

The breast cancer and heart disease messages were created based on content from the Canadian Breast Cancer Foundation and the Heart and Stroke Foundation of Canada. The messages were as similar as possible across the diseases and behaviors and were 155–165 words each. Each message started with the same definition of modifiable risk factors: “*Modifiable risk factors for heart disease are factors that can be changed. These are factors that we have more influence over and can affect us throughout our lives. Modifiable risk factors include behaviors such as physical activity, drinking alcohol, and smoking. We are able to influence our level of exposure to many of these factors. By learning about them, you can take steps to reduce your heart disease risk.*” This definition was followed by information about how and why physical activity and a healthy body weight are related to heart disease or breast cancer, and some risk statistics. For example, the physical activity and breast cancer message included: “*Regular physical activity helps improve your overall physical, emotional and social health and well-being. Another important reason to get more active is that this can lower your risk of breast cancer by as much as 25–30 per cent.*” The messages were shown on a computer and participants read them at their own pace.

### Measures

#### Automatically activated beliefs

As outlined by Huang and Hutchinson [8], four statement pairs (eight statements total) were presented in a random order with a final word or phrase missing. After 1000 milliseconds the last words appeared (the underlined sections in the statements below) and the participants agreed or disagreed as quickly as possible by pressing a key on a computer keyboard. Participants then gave a confidence rating for their response on a 3-point scale: maybe/‌probably/‌certainly. There were two variants of the task: one with breast cancer-related statements after reading the breast cancer messages, and the other with heart disease-related statements after reading the heart disease messages. The statements in both tasks were identical with the exception of the disease name. Within each variant (breast cancer or heart disease), one of the statement pairs was designed to capture responses to information about physical activity as preventive of the disease (My risk of breast cancer/heart disease is decreased/increased by being active). One captured overall perception of risk (Breast cancer/heart disease is/is not a real risk for me). Another pair of statements assessed automatic beliefs regarding if women who get the disease are like them (Women with breast cancer/heart disease are/are not like me). The final statement captured belief that women are to blame for getting the disease (Women with breast cancer/heart disease are/are not to blame).

#### Explicit believability

The statement “The messages related to breast cancer/heart disease are…” was rated on a 1–7 point scale with 8 adjective pairs such as believable/unbelievable, convincing/unconvincing, conclusive/inconclusive, and trustworthy/untrustworthy [3, 4]. The internal reliability was high for the breast cancer messages, α = .89, and the heart disease messages, α = .90. Items were reverse scored and the mean calculated so that a higher score indicates greater believability.

#### Message involvement

The statement “The messages related to breast cancer/heart disease are…” was rated on a 1–7 point scale with 8 adjective pairs such as unimportant/important and irrelevant/relevant [12]. The internal reliability was high for breast cancer message involvement, α = .85, and heart disease message involvement, α = .84.

#### Attitude

Affective attitude toward physical activity was assessed with two items on a 1–7 scale with the pairs: pleasant – unpleasant, and enjoyable- unenjoyable. These items were highly correlated, *r* = .80, and a mean of reverse scored items was calculated such that a higher score represents higher affective attitude.

#### Intentions

Intention to engage in physical activity was assessed with one question: “I intend to be physically active for at least 150 minutes a week” on a 1 (extremely unlikely) to 7 (extremely likely) scale.

#### Leisure-time physical activity (LTPA)

Self-reported LTPA was assessed with an item validated by Johansson and Westerterp [15] using doubly-labelled water. Participants responded to the statement: ‘Describe your physical activity at leisure time. If the activities vary between summer and winter, try to give an estimate for the average year round’. Response options were: very light (almost no activity at all), light (light activity approximately once a week - e.g., walking, nonstrenuous cycling or gardening), moderate (regular activity several times a week - e.g., walking, bicycling, gardening or walking to work 10–30 min a day), active (regular activities that cause you to breathe a bit more heavily more than once a week - e.g., intense walking or cycling), or very active (strenuous activities several times a week - e.g., running or sports).

#### Demographics

Participants reported their age (in years), height and weight (used to calculate body mass index [BMI]), smoking status, highest level of education, marital status, household income (in increments), and ethnicity (open-ended). Participants also reported if they had ever been diagnosed by a doctor or nurse with high blood pressure, high cholesterol, heart disease, stroke, angina, diabetes, or cancer (and if yes to cancer, what kind).

### Procedure

All procedures were approved by a university health research ethics review panel and written informed consent was obtained. A within subjects’ design was used. Each participant read either the breast cancer or heart disease message first, then completed the corresponding implicit believability task and questionnaires. They then read the other disease messages and completed the corresponding measures. Order of message was randomly assigned to participants such that half read the breast cancer message first and half read the heart disease messages first.

#### Data analysis

A belief confidence index (BCI) was calculated for each of the statement pairs in the implicit believability measure using response (agree/disagree) and confidence, as advocated by Huang and Hutchinson ([8]; p. 112). The possible score range is − 6 to + 6; a score of + 6 indicates the highest confidence in agreeing that being physically active decreases risk for heart disease (while also having the highest confidence in disagreeing that being physically active increases risk of heart disease). A higher risk BCI score indicates stronger beliefs of the real risk for getting breast cancer or heart disease. A higher ‘like me’ BCI score indicates belief that women with the diseases are ‘like me’ and a higher blame BCI score indicates belief that women who get either disease are to blame. A series of repeated measures analysis of variance tests (RM ANOVAs) examined within subject differences in breast cancer compared to heart disease message involvement, explicit believability, physical activity BCI, risk BCI, like me BCI, and blame BCI with LTPA group as the between subjects factor. Two multinomial regression models (breast cancer and heart disease) were used to examine intentions, attitudes, and disease specific message involvement, explicit believability, and BCI scores (physical activity as preventive, real risk, like me, and blame), as possible predictors of physical activity groups.

## Results

### Demographics

Incomplete data from three participants were not used, and one participant diagnosed with heart disease and breast cancer was also omitted from the analysis, leaving a final sample of 96 who ranged in age from 18 to 80 years. Demographic data are reported in Table 1. Ten participants chose not to answer the income question and one did not report marital status. One participant reported very light activity, 22 reported light activity, 27 reported moderate activity, 34 reported being active, and 12 reported being very active. Given the small numbers in the very light and very active groups, the very light and light active groups were combined to create an inactive LTPA group (*n* = 23), the moderately active group remained at *n* = 27, and the active and very active groups were combined to create an active LTPA group (*n* = 46). There were no differences between order of reading and measures (i.e., breast cancer reading and measures first compared to heart disease reading and measures first) in any demographic variable, nor in involvement with the heart disease or breast cancer messages, heart disease or breast explicit believability or physical activity belief confidence index, affective attitudes, intentions, or LTPA group (all *p* > .10). There was also no difference in age, *p* = .35, BMI, *p* = .55, nor smoking status, *p* = 49, by LTPA group. Three smokers were inactive, one was moderately active, and four reported being active.

**Table 1:** Participant demographics

Demographic variable		
Age *M* (SD)		32.3 (15.29)
BMI *M* (SD)		23.3 (5.05)
Smokers *N* (%)		8 (8.3%)
Education *N* (%)	High school or some college	12 (12.6%)
	College or technical school	14 (14.6%)
	Bachelor’s degree or higher	70 (72.9%)
Income *N* (%)	< $20,000/year	17 (17.7%)
	$20,000 - $39,999/year	17 (17.7%)
	$40,000 - $59,000/year	15 (15.6%)
	$60,000 - $79,999/year	19 (19.8%)
	$80,000 - $99,999/year	7 (7.3%)
	> $100,000/year	11 (11.5%)
Marital status *N* (%)	Single	59 (61.5%)
	Married	33 (34.4%)
	Divorced/widowed	3 (3.1%)
Ethnicity	White/Caucasian	34 (35%)
	Asian or Chinese	23 (23.7%)
	Indian	12 (12.4%)
	Other (e.g., Arab, Filipino, Iranian, Black)	28 (28.9%)

### Differences between breast cancer and heart disease message involvement, explicit believability, physical activity BCI, risk BCI, like me BCI, and blame BCI

Table 2 shows the means (SD) for all predictors in the models and the correlations between constructs. There was no difference in message involvement with breast cancer compared to heart disease messages, F (1, 93) = 1.81, *p* = .18, nor was there a difference in message involvement by LTPA group, F (2, 93) = .71, *p* = .50. There was also no difference in explicit believability of the messages, F (1, 93) = 1.83, *p* = .18, nor was there a difference by LTPA group, F (2, 93) = .18, *p* = .83. There was no overall difference in breast cancer compared to heart disease physical activity BCIs, F (1, 93) = 1.07, *p* = .30, but there was a difference by LTPA group, F (2, 93) = 3.90, *p* = .02. Post hoc tests showed no differences in the inactive or moderately active groups but the active group had a higher heart disease physical activity BCI than breast cancer physical activity BCI, *p* = .01, Cohen’s d = 0.54. There was a significant difference in risk BCIs, F (1, 93) = 9.54, *p* = .003, Cohen’s d = 0.27, but no difference by LTPA group, F (2, 93) = 1.12, *p* = .33. There was no difference in BCIs that women with breast cancer are ‘like me’ compared to women with heart disease are ‘like me’, F (1, 93) = 2.10, *p* = .15, and no difference by LTPA group, F (2, 93) = 0.99, *p* = .38. There was a significant difference in blame BCIs, F (1, 93) = 32.52, *p* < .001, Cohen’s d = 0.67, but no difference by LTPA group, F (2, 93) = 1.21, *p* = .30.

**Table 2:** Means (SD) and correlations between constructs; breast cancer correlations are above the diagonal and heart disease correlations are below the diagonal

	M (SD) HD	PA Intention	PA Attitude	Message Involvement	Explicit Believability	PA BCI	Risk BCI	Like me BCI	Blame BCI
M (SD) BC		5.51 (1.94)	5.55 (1.58)	5.86 (0.95)	5.59 (1.02)	4.66 (2.28)	1.41 (4.01)	0.53 (4.21)	−3.75 (2.64)
PA Intention	5.51 (1.94)	1	.357	.048	.164	−.012	−.066	−.069	.030
PA Attitude	5.55 (1.58)	.357	1	.385	.415	.070	−.089	−.032	−.091
Message involvement	5.76 (0.95)	.022	.409	1	.559	.178	−.061	−.012	−.028
Explicit believability	5.70 (0.98)	.061	.343	.586	1	.103	−.173	−.051	.122
PA BCI	5.10 (2.06)	.087	.157	.136	−.067	1	−.096	−.232	.058
Risk BCI	0.26 (4.14)	−.168	−.123	−.065	.002	−.150	1	.347	−.142
Like me BCI	0.08 (4.14)	.056	−.205	−.033	−.036	−.263	.482	1	−.074
Blame BCI	−1.67 (3.47)	−.173	−.076	−.076	.091	−.144	.101	−.114	

*HD* heart disease, *BC* breast cancer, *PA* physical activity, *BCI* Belief confidence index

### Prediction of LTPA group

The multinomial regression model predicting physical activity group after reading the breast cancer messages is summarized in Table 3. The null hypothesis can be rejected but the model had poor fit; about 28% of the variance was accounted for. The likelihood ratio tests showed that only intentions and attitudes predicted LTPA group and removing breast cancer specific message involvement, explicit believability, and all BCIs would improve fit. The model was able to classify 65.2% of the inactive group and 84.8% of the active group, but only 3.7% of the moderately active group. Higher affective attitude was related to greater likelihood of being moderately active or active compared to inactive, and higher intention was related to greater likelihood of being active compared to inactive.

**Table 3:** Multinomial regression models predicting moderately active and active groups compared to the inactive group after reading messages

		Breast Cancer Model		Heart Disease Model	
Model Fit		Χ^2^ = 27.63 (df = 16)^*^		Χ^2^ = 46.23 (df = 16)^***^	
Goodness of fit		Pearson Χ^2^ = 240.51 (df = 174)^***^		Pearson Χ^2^ = 21.63 (df = 174)^*^	
Variance accounted for		Nagelkerke pseudo R-square = .285		Nagelkerke pseudo R-square = .435	
LTPA group	Predictor	Exp(B)	95% confidence interval	Exp(B)	95% confidence interval
Moderately Active	Intentions	1.33	.96–1.84	1.37	.97–1.94
	Affective attitudes	1.87^*^	1.14–3.06	1.60	.98–2.62
	Message involvement	0.68	.27–1.72	.91	.36–2.31
	Explicit believability	0.79	.34–1.88	1.03	.43–2.47
	Physical activity BCI	1.14	.85–1.51	1.48^*^	1.0–2.21
	Risk BCI	1.04	.87–1.23	.95	.80–1.14
	Like me BCI	1.04	.88–1.22	1.10	.89–1.36
	Blame BCI	1.26	.95–1.66	1.05	.86–1.28
Active	Intentions	1.58^**^	1.14–2.18	1.61^**^	1.13–2.31
	Affective attitudes	1.88^**^	1.17–3.01	1.98^**^	1.20–3.28
	Message involvement	0.58	.24–1.38	0.41	.15–1.12
	Explicit believability	0.89	.39–2.04	1.62	.66–4.00
	Physical activity BCI	1.12	.86–1.45	2.59^**^	1.51–4.44
	Risk BCI	1.04	.89–1.22	1.00	.83–1.20
	Like me BCI	1.04	.90–1.21	1.24^*^	1.00–1.54
	Blame BCI	1.25	.95–1.63	1.11	.91–1.35

*BCI* Belief Confidence Index

^*^p < .05, ^**^ *p* < .01, ^***^ *p* < .001

The model predicting physical activity group after reading the heart disease messages is summarized in Table 3. The null hypothesis can be rejected but the model did not fit well; about 43% of the variance was accounted for. The likelihood ratio tests showed that removing explicit believability, and the risk and blame BCIs would improve fit. The model was able to classify 60.9% of the inactive group, 29.6% of the moderately active group, and 80.4% of the active group. Higher affective attitudes and intentions were related to greater likelihood of being active compared to inactive. The additional variance in this model could be accounted for by the physical activity and ‘like me’ BCIs. Each point increase in the BCI that being physically active reduces risk of heart disease was related to 1.48 times greater likelihood of being moderately active or 2.59 times greater likelihood of being active compared to inactive. Further, each point increase in the ‘like me’ BCI meant a 1.24 times greater likelihood of being in the active group compared to the inactive group.

## Discussion

This research examined message involvement, and the automatically activated and explicit believability of messages about breast cancer and heart disease prevention, and the relationships of these variables, in conjunction with physical activity related attitudes and intentions, to self-reported physical activity behavior. The correlations showed that, in partial support of the first hypothesis, involvement with both the breast cancer and heart disease messages was related to explicit believability of the respective messages. However, contrary to the hypothesis, message involvement was not related to any BCI score. O’Cass and Griffin [4] also reported relationships between message involvement and explicit believability. The findings of the present research demonstrate that message specific beliefs, operationalized through BCI scores, are independent of explicit believability (which is a much broader assessment of believability and trustworthiness) and message involvement. The findings corroborate Huang and Hutchinson’s [8] contention that the task they developed is able to provide more nuanced information about specific message-related thoughts, thus providing more information about message effects.

Intention to be active was not related to any variable other than attitudes. Attitude toward physical activity was, however, positively correlated with both breast cancer and heart disease message involvement and explicit believability, again supporting relationships reported by O’Cass and Griffin [4]. Even though affective attitudes were measured (i.e., how pleasurable they find physical activity), higher attitudes were related to higher feelings that the messages meant something to them, were relevant and important, and that the messages were trustworthy and convincing. Attitude was not strongly related to any BCI score for either disease. Huang and Hutchinson [8] argue that measures of explicit believability will be more related to attitudes when attitudes are based on more accessible beliefs; the results of the present research indicate that believing physical activity is a preventive behavior is likely easily accessible. Correlations between belief confidence indices further showed that participants who endorsed the belief that being physically active will help reduce risk of breast cancer or heart disease, had lower confidence in their beliefs that women with the diseases are ‘like me’. Further, both disease ‘like me’ BCIs were positively related to corresponding disease risk BCIs.

Participants, regardless of their self-reported activity level, were equally involved with the breast cancer and heart disease messages and expressed equally high explicit believability of the messages. There was also no difference in belief confidence that women ‘like me’ are at risk for breast cancer comparted to heart disease. These results do not support the hypothesis that more active women will have higher scores for these constructs. However, active participants had higher heart disease compared to breast cancer physical activity BCI scores. This indicates that active participants were more likely confident in their belief that being physically active will help reduce risk of heart disease compared to breast cancer. There is likely greater internalization of the message that physical activity can help prevent heart disease by already active women and the messages may reinforce already held beliefs. However, it is not known if the participants are active because they are, in part, aware that being active will reduce risk of heart disease.

There was also greater belief of being at risk of breast cancer compared to heart disease, and of perceptions of blame, regardless of LTPA group. Although the effect was small, women felt more at risk for breast cancer, supporting previous research that explicitly assessed risk perceptions of these diseases [10]. Regarding the belief that women who develop one of these diseases are to blame, the average blame BCIs were negative indicating that, in general, the participants did not believe that women who get the diseases are to blame. However, this effect was stronger for breast cancer and the standard deviation for heart disease was much larger indicating more variability in this construct across participants. This may be related to the sense of control some women have over heart disease in comparison to breast cancer [10].

In support of the fourth hypotheses, there were stronger relationships between heart disease message constructs and LTPA group than there were for breast cancer. None of the breast cancer related BCIs increased prediction of activity group. Conversely, confidence in the belief that being active can reduce risk of heart disease was positively related to physical activity behavior. Also, greater confidence that women with heart disease are ‘like me’ was related to being more active, compared to inactive. The differences between the two disease-specific multinomial regression models may be a function of how the diseases are discussed in the media: heart disease is presented as avoidable through preventive behaviors whereas breast cancer is something one survives [11]. Researchers have also reported that women feel heart disease is more preventable than breast cancer [10]. This may result in memory associations between prevention behaviors such as physical activity and heart disease but not breast cancer. Understanding of the relationship between primary prevention and breast cancer is relatively recent. Although a 2017 review by the World Cancer Research Fund International [16] cited strong evidence of the relationship between physical activity and decreased risk of breast cancer, physical activity has been discussed as preventive of heart disease for far longer than it has been discussed as preventive of breast cancer. There is a need to create more accessible beliefs regarding the relationship between physical activity and breast cancer prevention. Although the participants in the present study report explicit believability of the breast cancer and heart disease messages, there may not be a strong enough memory trace to automatically activate associations between breast cancer and physical activity. Calitri and colleagues [12] report that among active people, only those who had a strong explicit attitude also showed attentional bias toward physical activity related stimuli, which they argue demonstrates the need for strengthening automatic associations and finding ways to direct attention. This may happen over time if breast cancer is discussed as preventive of physical activity to the same extent that heart disease is, but testing strategies such as evaluative conditioning within interventions may offer a way to shortcut the process [17].

This research has several strengths including a randomized within-subject experimental design with strong measures. However, although less error variance existed between messages because participants responded to both disease messages, the within subjects’ design may also have resulted in carryover effects from one reading to the next, such that reading about one disease may have influenced responses to the subsequently read message. This was somewhat mitigated by counterbalancing the reading order. Another limitation is that although efforts were made to recruit a diverse sample of women, participants were younger and for the most part highly educated. These demographic factors might influence perceptions of disease risk and message processing and the results of this study should be replicated with a more diverse sample of women. An additional consideration is the labelling of the task developed by Huang and Hutchinson [8] as an implicit measure. Although these authors argue that their task is implicit because participants are either unaware of its intention or responses are not easily controllable, it is possible that participants considered their responses before indicating their confidence in their agreement or disagreement with the statements. Nonetheless, the information provided by this task does provide more specific information about diverse content within the messages and highlights that various beliefs in relation to the messages differ from each other and can have different predictive effects.

## Conclusions

This research is an important contribution that highlights that active women hold stronger beliefs that physical activity is preventive of heart disease compared to breast cancer. It seems likely that prevention messages created by health promoters are processed differently by already active women who may have internalized the messages, making them more readily available within memory. Further, the more participants indicated that women with heart disease are ‘like me’ the more likely they were to be active. This may demonstrate internalization of the idea that one is at risk for heart disease and that this may be related to decisions to be active. These results highlight that health promoters may be at risk of reaching those already engaged in prevention behaviors. Finding innovative ways of reaching those who are not interested in physical activity is needed. For example, investigating if inactive women find prevention information threatening is warranted. Other researchers have reported that healthy women who were moderate alcohol drinkers automatically avoided cancer-related words after being informed that alcohol is a risk factor for breast cancer, but self-affirmation moderated this relationship [18]. It is likely women who were not self-affirmed found the information threatening. Further, the current research showed that breast cancer prevention messages are likely not as strongly internalized as heart disease prevention messages. Women fear breast cancer and greater understanding of how to mitigate these fears, and how these fears are related to responses to prevention messages, is a rich area of study that may contribute to more effective health promotion.

## Abbreviations

BCI: belief confidence index
